# Genome-wide identification and characterization of *GRAS* genes in soybean (*Glycine max*)

**DOI:** 10.1186/s12870-020-02636-5

**Published:** 2020-09-05

**Authors:** Liang Wang, Xianlong Ding, Yingqi Gao, Shouping Yang

**Affiliations:** Soybean Research Institute, National Center for Soybean Improvement, Key Laboratory of Biology and Genetic Improvement of Soybean (General, Ministry of Agriculture), State Key Laboratory of Crop Genetics and Germplasm Enhancement, Jiangsu Collaborative Innovation Center for Modern Crop Production, College of Agriculture, Nanjing Agricultural University, Nanjing, 210095, China

**Keywords:** Soybean, *GRAS*, Genome-wide, Evolutionary analyses, Expression patterns, Saline and dehydration stresses, Seed germination

## Abstract

**Background:**

GRAS proteins are crucial transcription factors, which are plant-specific and participate in various plant biological processes. Thanks to the rapid progress of the whole genome sequencing technologies, the *GRAS* gene families in different plants have been broadly explored and studied. However, comprehensive research on the soybean (*Glycine max*) *GRAS* gene family is relatively lagging.

**Results:**

In this study, 117 *Glycine max* *GRAS* genes (*GmGRAS*) were identified. Further phylogenetic analyses showed that the *GmGRAS* genes could be categorized into nine gene subfamilies: DELLA, HAM, LAS, LISCL, PAT1, SCL3, SCL4/7, SCR and SHR. Gene structure analyses turned out that the *GmGRAS* genes lacked introns and were relatively conserved. Conserved domains and motif patterns of the *GmGRAS* members in the same subfamily or clade exhibited similarities. Notably, the expansion of the *GmGRAS* gene family was driven both by gene tandem and segmental duplication events. Whereas, segmental duplications took the major role in generating new *GmGRAS* genes. Moreover, the synteny and evolutionary constraints analyses of the GRAS proteins among soybean and distinct species (two monocots and four dicots) provided more detailed evidence for *GmGRAS* gene evolution. *Cis*-element analyses indicated that the *GmGRAS* genes may be responsive to diverse environmental stresses and regulate distinct biological processes. Besides, the expression patterns of the *GmGRAS* genes were varied in various tissues, during saline and dehydration stresses and during seed germination processes.

**Conclusions:**

We conducted a systematic investigation of the *GRAS* genes in soybean, which may be valuable in paving the way for future *GmGRAS* gene studies and soybean breeding.

## Background

The GRAS transcription factors (TFs) are plant-specific regulating proteins that have been widely studied in the past decade [1–4]. The name of GRAS proteins was derived from the three first identified members of the gene family: gibberellic acid insensitive (GAI), repressor of GA1–3 mutant (RGA), and scarecrow (SCR) [5]. In general, the GRAS protein sequences consisted of 400–770 amino acid residues, which exhibited highly conserved C-terminal regions and variable N-terminals [6, 7]. Commonly, the GRAS domains were determined by the conserved carboxyl-terminal regions, and could be divided into five motifs: leucine-rich region I (LHRI), VHIID, leucine-rich region II (LHRII), PFYRE, and SAW. Notably, these five motifs played important roles in the interactions between GRAS with other proteins [8]. According to the early research, LHRI and LHRII were crucial for the homologous dimerization of GRAS proteins. The VHIID motif was the core component of the GRAS protein, which contained a very conserved P-N-H-D-Q-L unit and ended with L-R-I-T-G. Three pairs of conserved protein sequence characters, P, FY, and RE, could be recognized and assembled into the PFYRE motif, which might be correlated to phosphorylation. And the SAW motif consisted of three conserved amino acid residues: R-E, W-G, and W-W [4, 6, 9]. By contrast, the fickle N-terminus of GRAS proteins could be folded and modified into specific molecular binding structures. Based on these, the GRAS proteins broadly participated in many critical processes such as signal transductions, root radial elongations, axillary shoot meristem formations and stress responses in plants [10–13]. Previously, the *GRAS* gene family in *Arabidopsis thaliana* was separated into eight subfamilies, including DELLA, SCL3, LAS, SCR, HAM, SHR, LISCL and PAT1 [14]. The DELLA subfamily contained the *GAI*, *RGA* and *RGL* genes, and was reported as the main repressors of gibberellin signal transduction [15]. Importantly, the SCL3 proteins were validated as the switches of mediating the elongation of the root [16]. Moreover, the SCL3 proteins could cooperate with the DELLA proteins and adjusted gibberellin feedback via IDD proteins [17]. Besides, SHR and SCR proteins tended to form the SCR/SHR complex, which was determined to be associated with root radial patterning [18, 19]. LAS proteins were reported tightly linked to the lateral shooting formation during the vegetative growth stages of Arabidopsis [11]. Furthermore, the overexpression of *VaPAT1* (a *GRAS* gene of *Vitis amurensis*) improved the abiotic stress tolerance in the transgenic Arabidopsis [20]. Another study turned out that *AtSCL13* (a member of the PAT1 subfamily in *Arabidopsis thaliana*) involved in phytochrome A (phyA) signal transduction and played a major role in hypocotyl elongation [21]. In *Medicago truncatula*, the HAM subfamily gene *MtNSP2* together with the *MtNSP*1 (the SHR subfamily gene), formed a DNA binding complex to induce gene expression during nodulation signaling [22]. In Petunia, the *PhHAM* genes acted on adjacent tissues in noncellular autonomous ways and maintained the activities of the apical meristem [23]. With the rapid development of sequencing technologies, several new subfamilies, for instance, DLT, SCL4/7, Os19, Os4 and PT20, gradually enriched the former *GRAS* gene subfamilies in diverse plants [24]. To date, there are over 30 mono- and dicotyledonous plants, such as rice, maize, Arabidopsis, cotton, *Malus domestica* and castor beans have been carried out genome-wide *GRAS* gene family identifications and analyses [3, 8, 19, 25, 26].

Soybean (*Glycine max* L.) is one of the major crops abundant in high-quality protein and oil, which also contains various nutrients such as lecithins and isoflavones [27]. Many soybean transcription factor families like WKRY [28], MYB [29], NAC [30], HD-Zip [31], ARF [32] and MADS [33] have been investigated and studied. However, comprehensive studies on the *Glycine max GRAS* gene (*GmGRAS*) family are relatively lagging. Owing to the importance of the *GRAS* genes in plant developmental and physiological courses, it is imperative to conduct relevant explorations and analyses to fix the gap. In this study, we systematically identified 117 *GmGRAS* gene members from the soybean genome. First, we investigated the phylogenetic relations, gene structures, motif compositions, chromosomal locations and gene duplication events of the identified GmGRAS members. Next, we carried out the evolutionary analyses on the GRAS members among soybean and four dicotyledons (*Arabidopsis thaliana*, *Glycine soja*, *Vigna unguiculata* and *Solanum lycopersicum*) as well as two monocotyledons (*Oryza sativa* and *Sorghum bicolor*). Moreover, we analyzed *cis*-elements in promoter regions of the *GmGRAS* genes. Besides, we explored the expression patterns of the *GmGRAS* genes in different tissues, during saline and dehydration stresses and during seed germination processes. In particular, due to the importance of seed germination in soybean production, 18 representative soybean *GRAS* genes were further selected and carried out the quantitative RT-PCR analyses. Collectively, the current research provided insights for the future functional study of *GmGRAS* genes and may be valuable for soybean breeding.

## Results

### Identification of *GmGRAS* genes in soybean

Totally 117 *GmGRAS* genes were identified from the soybean *Wm82.a2.v1* genome on Phytozome (https://phytozome.jgi.doe.gov/pz/portal.html#). Among them, 116 genes were mapped on the 20 different soybean chromosomes and one gene (*Glyma.U013800.1.Wm82.a2.v1*) was located on unattributed scaffold_21 of the soybean genome, which was renamed as *GmGRAS117*. According to the chromosome names and chromosomal locations, the rest 116 *GmGRAS* genes were renamed from *GmGRAS1* to *GmGRAS116*, respectively (Additional file 1: Table S1).

The basic characteristics of GmGRAS family members were listed in Table S1 (Additional file 1), including the open reading frame (ORF) length, the protein size, the protein molecular weight (MW), isoelectric point (pI), the predicted subcellular localization, the putative conserved domain, homologs in other species. As is shown in Table S1 (Additional file 1), GmGRAS55 was the smallest protein with 169 amino acids (aa), whereas the largest one was GmGRAS111 (843 aa). The MW of the proteins spanned from 18,975.84 to 91,543.91 Da, and the pI ranged from 4.76 (GmGRAS33) to 9.21 (GmGRAS55). The predicted subcellular localization results showed that 74 GmGRAS proteins were located in the nuclear region, 29 in the cytoplasm, ten in the plasma membrane, two in the extracellular region, one in the chloroplast, and one in the mitochondria. And the coding sequences and the protein sequences of the identified *GmGRAS* gene members were listed in Table S2 (Additional file 2).

### Phylogenetic analyses and classifications of *GmGRAS* gene members

**Fig. 1:**
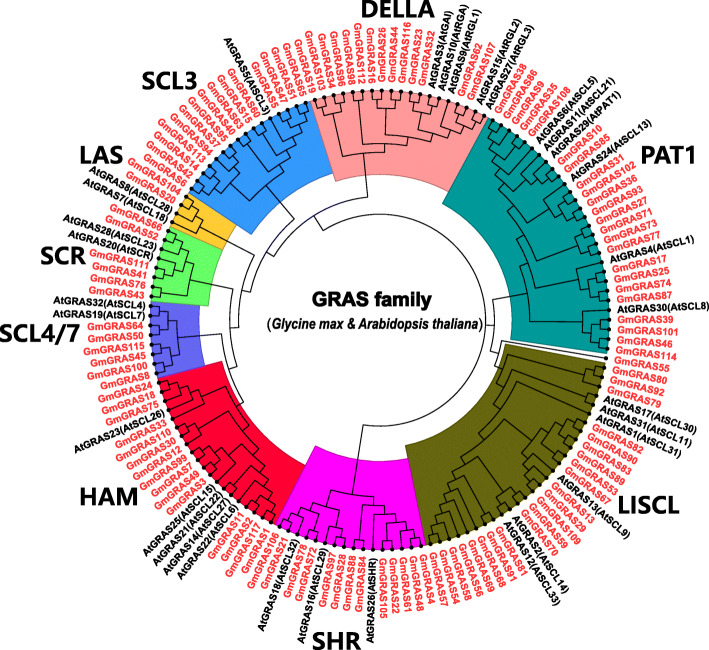
Unrooted phylogenetic tree of GRAS proteins in soybean and Arabidopsis. The GRAS protein sequences of the two species were aligned by MEGA 7.0 with the MUSCLE method, and the tree was built with the neighbor-joining (NJ) method. The tree was further categorized into nine distinct subfamilies in different colors. All the GmGRAS proteins have been emphasized in red

To classify the phylogenetic relationships of soybean GRAS proteins, we constructed a phylogenetic tree based on the identified 117 GmGRAS in this study and 32 reported Arabidopsis GRAS proteins from TAIR (https://www.arabidopsis.org/index.jsp) (Additional file 3: Table S3). The phylogenetic analyses showed that the 116 *GmGRAS* gene members were divided into nine subfamilies: DELLA, HAM, LAS, LISCL, PAT1, SCL3, SCL4/7, SCR, SHR. Comparably, GmGRAS55 was relatively independent, which did not belong to any *GRAS* gene subfamilies (Fig. 1). As is shown in Fig. 1 and Table S1 (Additional file 1), the PAT1 subfamily herein contained 23 members and was the largest *GmGRAS* gene subfamily in this study. The LISCL subfamily was one gene member less than the PAT1 subfamily. Coincidentally, both the HAM and SCL3 subfamilies included 15 members. Besides, there were 14, 13, 6, 6 and 2 *GmGRAS* gene members in the DELLA, SHR, SCR, SCL4/7 and LAS subfamilies, respectively.

### Gene structures and motif patterns of *GmGRAS* gene members

**Fig. 2:**
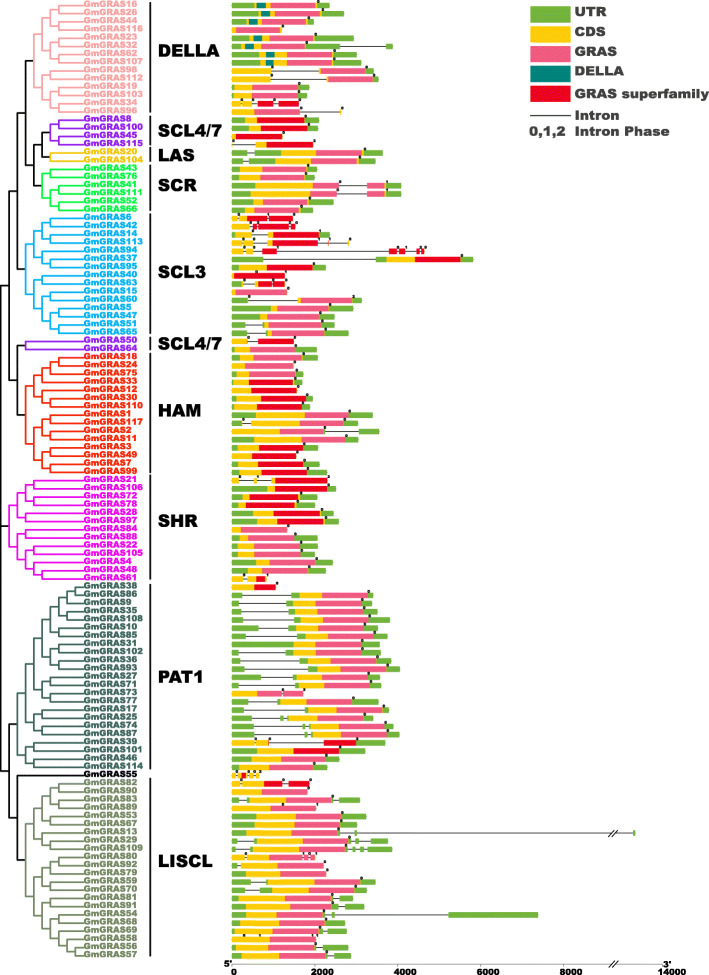
Phylogenetic clustering and gene structures of the GmGRAS members. Left panel: phylogenetic clustering of the GmGRAS members. The GmGRAS members were classified into nine subfamilies. Right panel: gene structures of the GmGRAS members. Green boxes indicated untranslated 5′- and 3′-regions; yellow boxes indicated exons; black lines indicated introns. The numbers (0, 1, 2) indicated the phases of corresponding introns. The GRAS-related domains (GRAS, DELLA and GRAS superfamily) are highlighted in pink, dark green and red, respectively

By screening the corresponding genomic DNA sequences and the annotation files, the exon-intron patterns of the identified *GmGRAS* genes were obtained. As is shown in Fig. 2, the *GmGRAS* genes displayed one to seven exons (91 with one exon, 14 with two exons, five with three exons, five with four exons, one with five exons, and one with seven exons) and lacked introns. For the protein conserved domains, all the 117 GmGRAS members possessed at least one GRAS or GRAS superfamily domain. Members in the same subfamily or clade have similar gene structures and protein conserved domains. For instance, GmGRAS16, GmGRAS23, GmGRAS26, GmGRAS32, GmGRAS44 and GmGRAS62 belonged to the DELLA subfamily, and each of them contained a DELLA protein domain with one exon and no intron (Fig. 2).

**Fig. 3:**
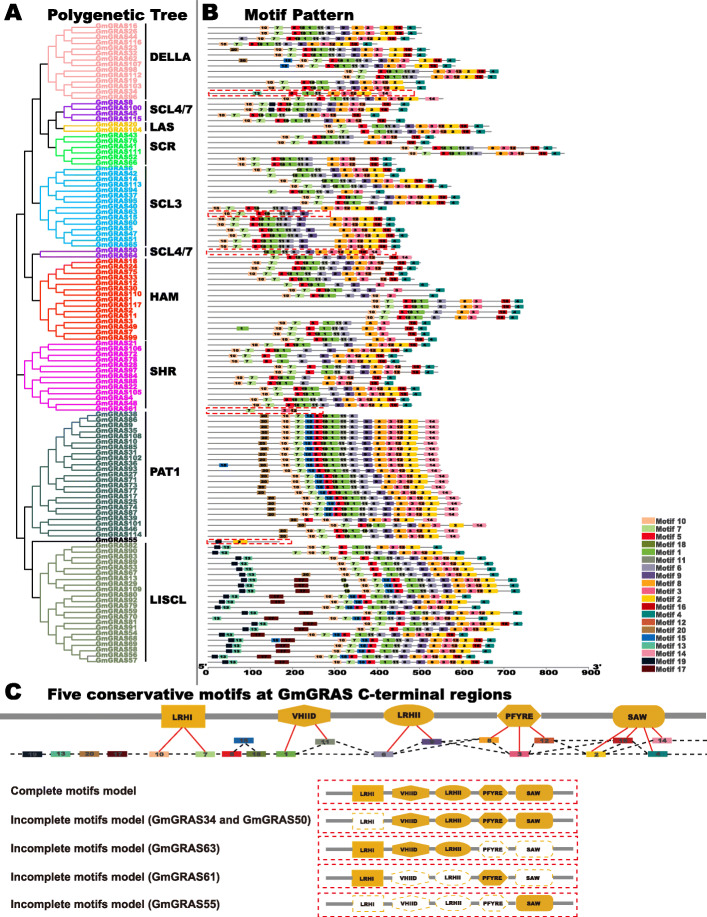
Phylogenetic clustering and the motif patterns of the GmGRAS members. **a** Phylogenetic clustering of the GmGRAS members. **b** Motif patterns of the GmGRAS members. The 20 distinct MEME-motifs were displayed in different colored boxes. The sequence information for each MEME-motif was provided in Table S4 (Additional file 4). The length of the protein can be estimated by using the scale at the bottom. **c** Schematic of five conservative motifs at the C-terminal regions of the GmGRAS members. The identified MEME-motifs were further classified into five conserved motifs: LHR I, VHIID, LHR II, PFYRE and SAW. The MEME-motifs components of the five conserved motifs were displayed in the top panel Fig. 3c. The incomplete five conserved motif sequences were noted with the red dashed boxes in Fig. 3b and depicted in the bottom panel of Fig. 3c

To further demonstrate the structures of the GmGRAS proteins, a schematic was built based on the MEME-motif scanning result. As is shown in Fig. 3b, 20 diverse MEME-motifs (named Motif 1–20) were displayed. Moreover, the details of these motifs were presented in Table S4 (Additional file 4) and the Seq Logos of the 20 MEME-motifs were exhibited in Fig. S1 (Additional file 5). Referring to the classifications of Quan et al. in *Juglans regia*, the MEME-motifs were further assessed and categorized into the five GRAS specific C-terminal motifs: LRHI, VHIID, LRHII, PFYRE and SAW [34]. As a result, Motifs 7 and 10 were classified into the LRHI motif; Motifs 1 and 11 belonged to the VHIID motif; Motifs 6 and 9 were associated with the LRHII motif; Motifs 3, 8 and 12 were included by the PFYRE motif; and Motifs 2, 4, 14 and 16 were in the SAW motif (Fig. 3b and Fig. 3c). Besides, Motifs 5, 15 and 18 were located between the LRHI and VHIID motifs. It is worth noting that the MEME-motifs in the five GRAS specific C-terminal motifs were not fixed. Sometimes, merely one MEME-motif existed in the C-terminal conserved motifs. However, some C-terminal conserved motifs were corresponding to two or three MEME-motifs. Interestingly, some soybean GRAS subfamilies contain unique MEME-motifs. For instance, the Motifs 13, 17 and 19 were only found in the LISCL subfamily. As is shown in Fig. 3b and Fig. 3c, most GmGRAS proteins contain the complete components of the five conservative motifs at C-terminals, however, with the exceptions that GmGRAS34, GmGRAS50, GmGRAS55, GmGRAS63 and GmGRAS61 lacked one to four subunits of the five conserved motifs (denoted with the red dotted boxes). Overall, the MEME-motifs in the specific *GmGRAS* gene subfamily or clade exhibited similar components and displayed orders.

### Chromosomal distributions, synteny and evolutionary analyses of *GmGRAS* gene members

**Fig. 4:**
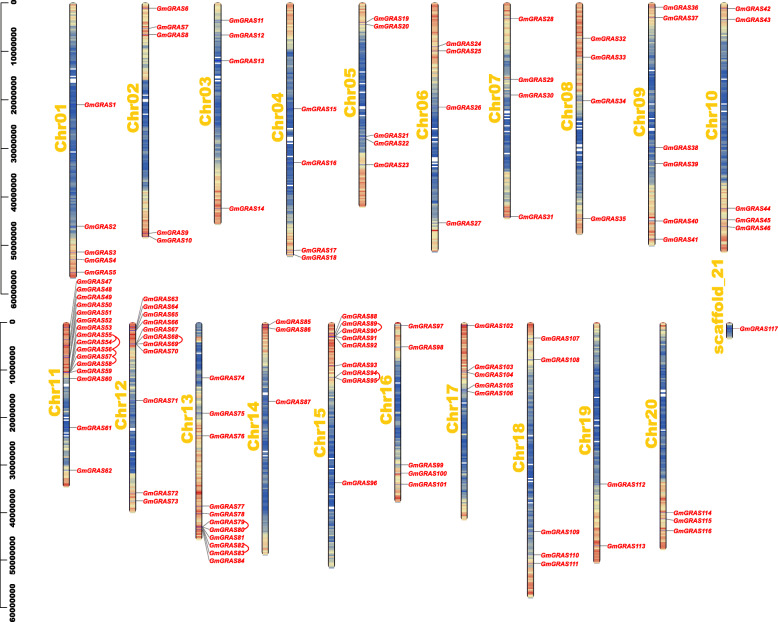
The chromosomal or scaffold distributions of the *GmGRAS* genes in the soybean genome. The red lines connected the tandem duplicated *GmGRAS* gene pairs. The chromosome or scaffold names were set at the left of the chromosomes or scaffold. The gene density of each chromosome or scaffold was evaluated by setting the genetic interval as 300-kb and was depicted by gradient colors from blue (low gene density) to red (high gene density). The blank regions on chromosomes or scaffold indicated that the genetic regions lacked gene distribution information

The *GmGRAS* gene chromosomal positions were depicted based on the gene physical location information of the soybean genome (Fig. 4). Importantly, the gene density of each chromosome or scaffold was also evaluated by setting the genetic interval as 300-kb in Table S5 (Additional file 6) and was further illustrated by gradient colors from blue (low gene density) to red (high gene density) in Fig. 4. The blank regions on chromosome or scaffold indicated that the genetic regions lacked gene distribution information. As is shown in Fig. 4, the 117 *GmGRAS* genes were unevenly distributed on the 20 soybean chromosomes (Chr01 – Chr20) and scaffold_21. And most identified *GmGRAS* genes tended to locate in the high gene density regions. Notably, Chr11 contained the most *GmGRAS* genes and 16 genes were located on this chromosome. Some chromosomes (e.g. Chr12, Chr13 and Chr15) have considerable *GmGRAS* gene members, whereas some (e.g. Chr19) have few, and there is only one *GmGRAS* gene on scaffold_21. Similar to the previous studies on *GRAS* genes in other species, no obvious correlation was found between the chromosome length and the number of *GmGRAS* genes [4, 7].

**Fig. 5:**
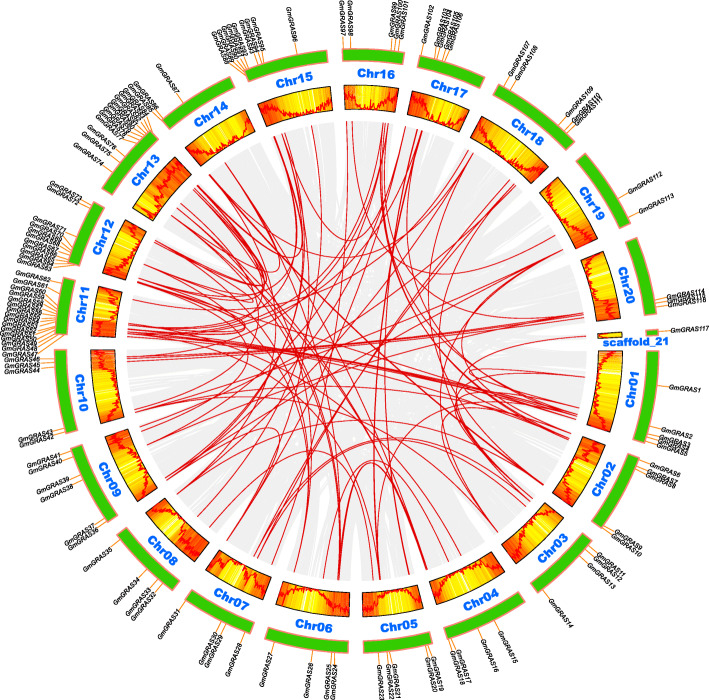
Inter-chromosomal relations of the *GmGRAS* genes in the soybean genome. All the syntenic blocks in the soybean genome were depicted by the gray lines, and the red lines linked the duplicated *GRAS* gene pairs. The gene density of 300-kb hereditary interval on each chromosome or scaffold was depicted by the heatmap and the wave graph

Early research demonstrated that gene duplications were essential for the occurrences of new gene functions and the expansions of the gene families [35]. Hence, we further explored the duplication events of the identified 117 *GmGRAS* genes (Fig. 4 and Fig. 5). In a previous study, Holub defined a tandem duplication event as a 200 kb (kilobase) intergenic region containing multiple (two or more) gene family members [36]. Comparably, segmental duplications frequently happened in plants because most plants are diploidized polyploids and retain numerous duplicated chromosomal blocks within their genome [37, 38]. And segmental duplications multiple genes through polyploidy followed by chromosome rearrangements [39]. Importantly, both segmental and tandem duplications were considered to be two representative main causes of gene family expansion in plants [37, 38]. In this study, fifteen *GmGRAS* genes were found in nine tandem duplication events (*GmGRAS54*/*GmGRAS55*, *GmGRAS55*/*GmGRAS56*, *GmGRAS56*/*GmGRAS57*, *GmGRAS57*/*GmGRAS58*, *GmGRAS68*/*GmGRAS69*, *GmGRAS79*/*GmGRAS80*, *GmGRAS82*/*GmGRAS83*, *GmGRAS89*/*GmGRAS90* and *GmGRAS94*/*GmGRAS95*) distributed on four soybean chromosomes (Chr11, Chr12, Chr13 and Chr15), which turned out these regions may be the hotspots for *GmGRAS* gene distributions (Additional file 7: Table S6). Notably, most tandem duplication events happened in the LISCL subfamily, except for *GmGRAS94*/*GmGRAS95*, which occurred in the SCL3 subfamily. Furthermore, 104 segmental duplication events associate with 107 *GmGRAS* genes were also detected (Fig. 5 and Additional file 7: Table S6). In summary, most *GmGRAS* genes possibly originated from the gene duplications, and the segmental duplication events may play a pivotal role in generating new *GmGRAS* genes.

**Fig. 6:**
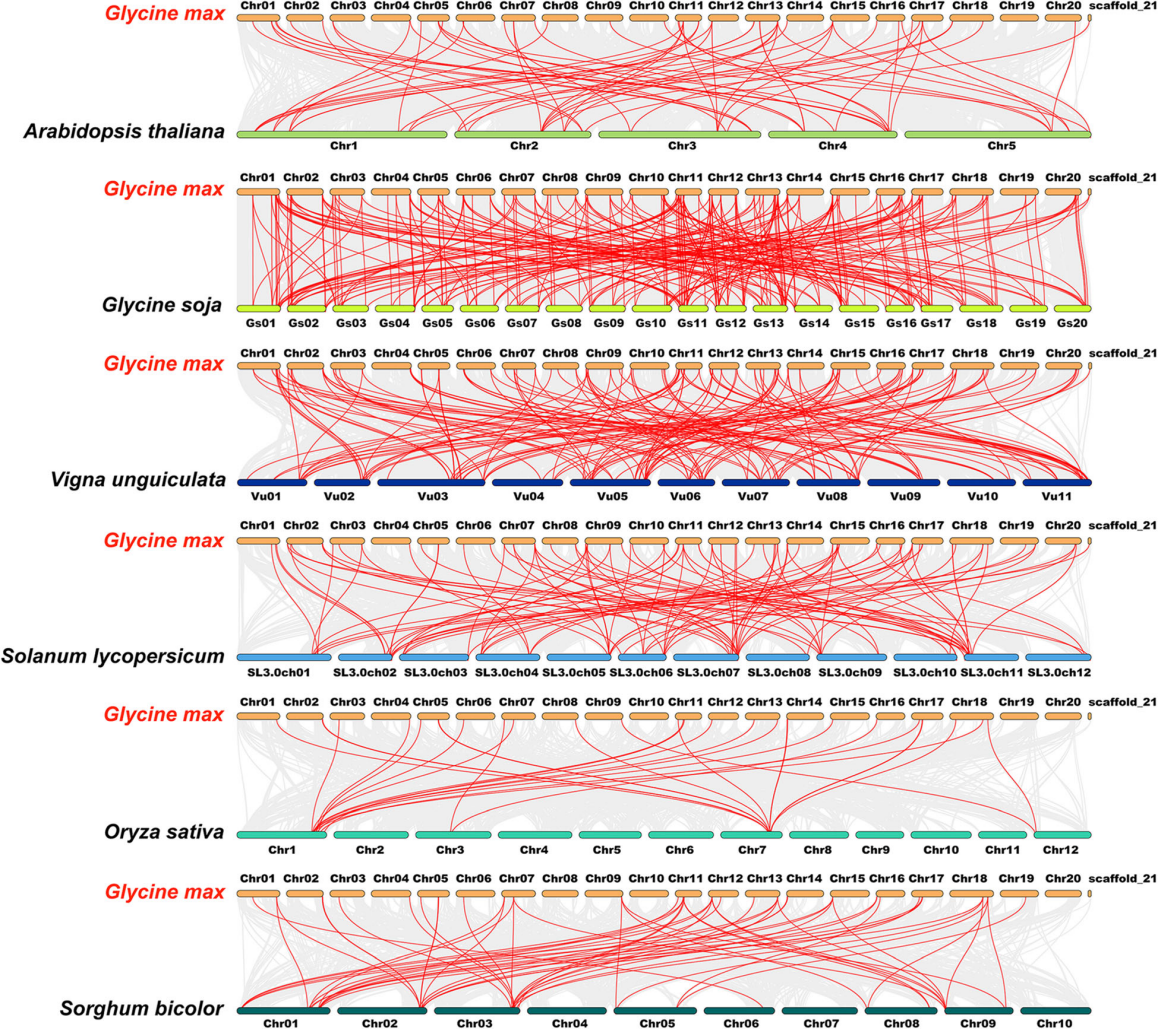
Synteny analyses of the *GRAS* genes between soybean and six representative species. The collinear blocks within soybean and other specie genomes were displayed by the gray lines. The syntenic *GRAS* gene pairs between soybean and other species were highlighted with the red lines

**Fig. 7:**
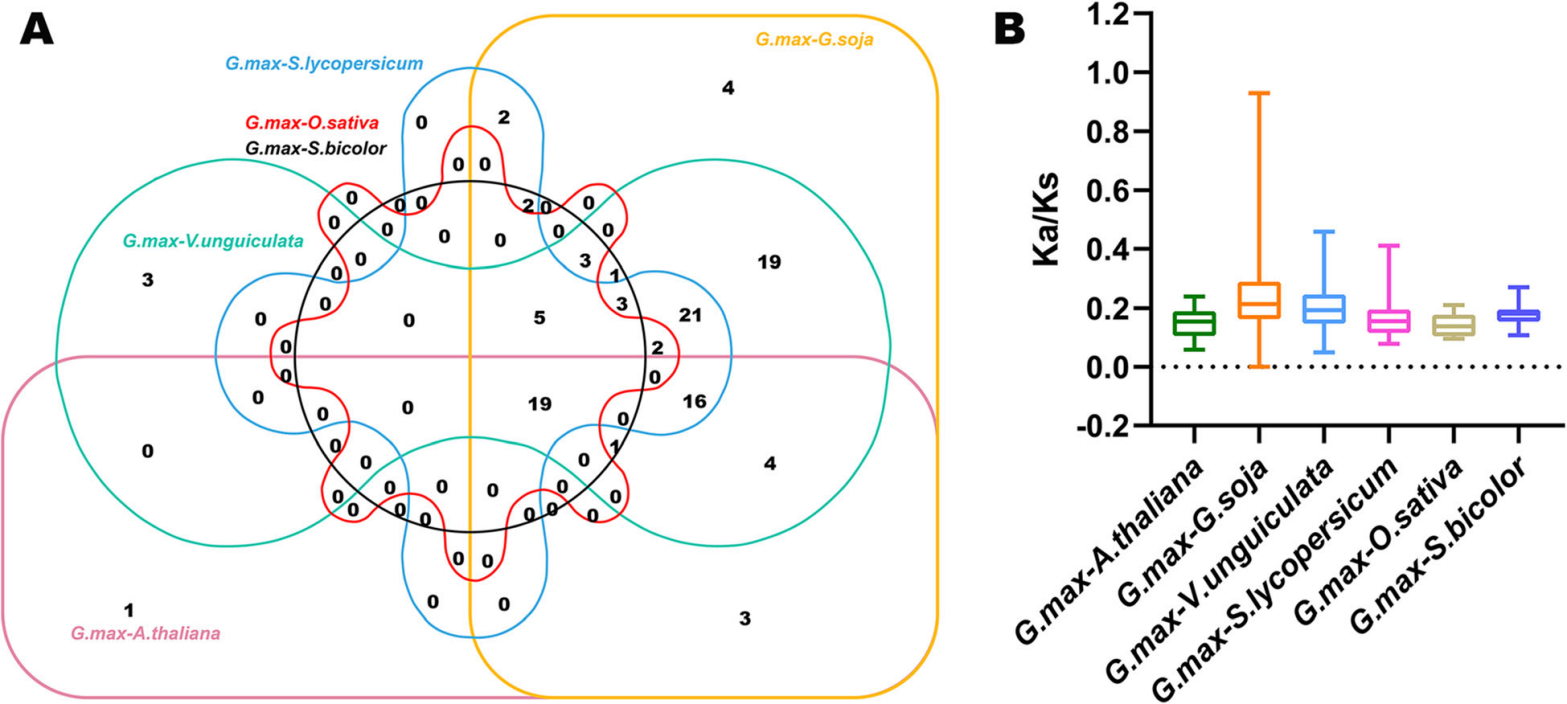
Non-redundant syntenic *GmGRAS* genes throughout diverse species and evolutionary analyses of the *GRAS* gene families. **a** The Venn diagram of syntenic *GRAS* genes throughout diverse species. **b** The ratio of nonsynonymous to synonymous substitutions (Ka/Ks) of *GRAS* genes in soybean and other six species. The species’ names with the prefixes ‘*G. max*’, ‘*A. thaliana*’, ‘*G. soja*’, ‘*V. unguiculata*’, ‘*S. lycopersicum*’, ‘*O. sativa*’ and ‘*S. bicolor*’ indicated *Glycine max*, *Arabidopsis thaliana*, *Glycine soja*, *Vigna unguiculate*, *Solanum lycopersicum*, *Oryza sativa* and *Sorghum bicolor*, respectively

To explore the evolutionary clues for the soybean *GRAS* gene family, we constructed six comparative syntenic graphs to display the synteny of *GRAS* gene members between soybean and six representative species (Fig. 6). The six representative species contained four dicots (*Arabidopsis thaliana*, *Glycine soja*, *Vigna unguiculata* and *Solanum lycopersicum*) and two monocots (*Oryza sativa* and *Sorghum bicolor*). Totally 109 *GmGRAS* gene members showed syntenic relationships with those in *Glycine soja* (101), *Vigna unguiculata* (49), *Solanum lycopersicum* (31), *Arabidopsis thaliana* (22), *Sorghum bicolor* (21) and *Oryza sativa* (9) (Additional file 8: Table S7). And the numbers of *GmGRAS* orthologous genes in *Glycine soja*, *Vigna unguiculata*, *Solanum lycopersicum*, *Arabidopsis thaliana*, *Sorghum bicolor* and *Oryza sativa* were 289, 162, 104, 64, 57 and 29, respectively. Overall, the *GmGRAS* genes consisted of more syntenic gene pairs with dicots compared to those in monocots. Furthermore, as the ancestor of *Glycine max* (soybean), *Glycine soja* exhibited superior synteny with soybean than the other five species. Importantly, as is shown in the interactive Venn diagram of *GRAS* genes throughout the different species (Fig. 7a), 19 *GmGRAS* genes had syntenic *GRAS* gene pairs in all the six species. And the 19 *GmGRAS* genes were highlighted in bold in Table S8 (Additional file 9). The syntenic gene pairs between soybean and other species may be valuable to illuminate the evolutions of *GRAS* genes.

In this study, the Ka/Ks (non-synonymous substitution/synonymous substitution) ratios of the *GmGRAS* orthologous gene pairs in the six species were calculated to evaluate the evolutionary constraints acting on soybean *GRAS* gene family (Additional file 8: Table S7). As is shown in Fig. 7b, all *GmGRAS* orthologous gene pairs displayed Ka/Ks < 1. Hence, we speculated that the soybean *GRAS* gene family might go through strong purifying selective pressures during the evolution [35].

### *Cis*-element analyses of soybean *GmGRAS* genes

**Fig. 8:**
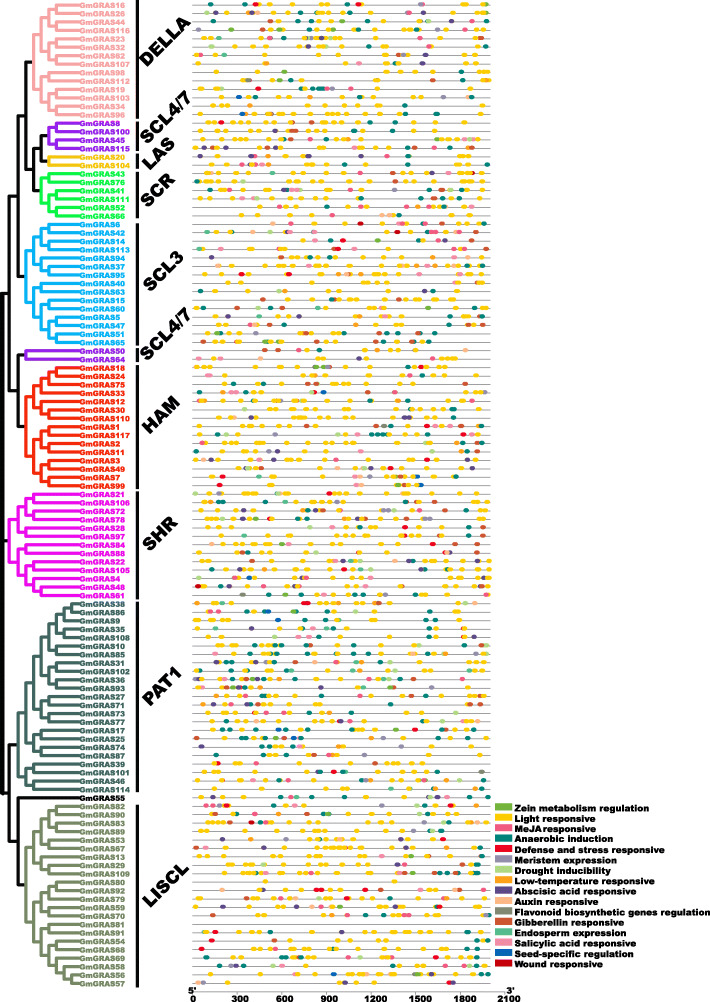
*Cis*-elements in the *GmGRAS* gene promoter regions. Left panel: phylogenetic clustering of the GmGRAS members. Right panel: the pattern of the *cis*-elements in the 2000 bp upstream hereditary regions of the identified *GmGRAS* genes. Different *cis*-elements were indicated by distinct colored round rectangles

The *cis*-elements play an essential role in transcriptional regulation of the gene expression [40]. In this study, the 2000-bp upstream sequences of the identified *GmGRAS* genes were extracted from the soybean genome, and the *cis*-element analysis was carried out by using PlantCARE (http://bioinformatics.psb.ugent.be/webtools/plantcare/html/) (Additional file 10: Table S9). As is displayed in Fig. 8 and Table S9 (Additional file 10), 16 types of *cis*-elements were acquired in 2000-bp promoter regions of the *GmGRAS* genes. Notably, the *cis*-elements like light responsive, auxin responsive, gibberellin responsive, abscisic acid responsive, MeJA responsive, defense and stress responsive, drought inducibility and anaerobic induction were broadly distributed, which indicated the *GmGRAS* gene diversely response to different abiotic stresses and regulate various biological processes.

### Expression profiles of *GmGRAS* genes in various tissues and gene expression correlation analyses

**Fig. 9:**
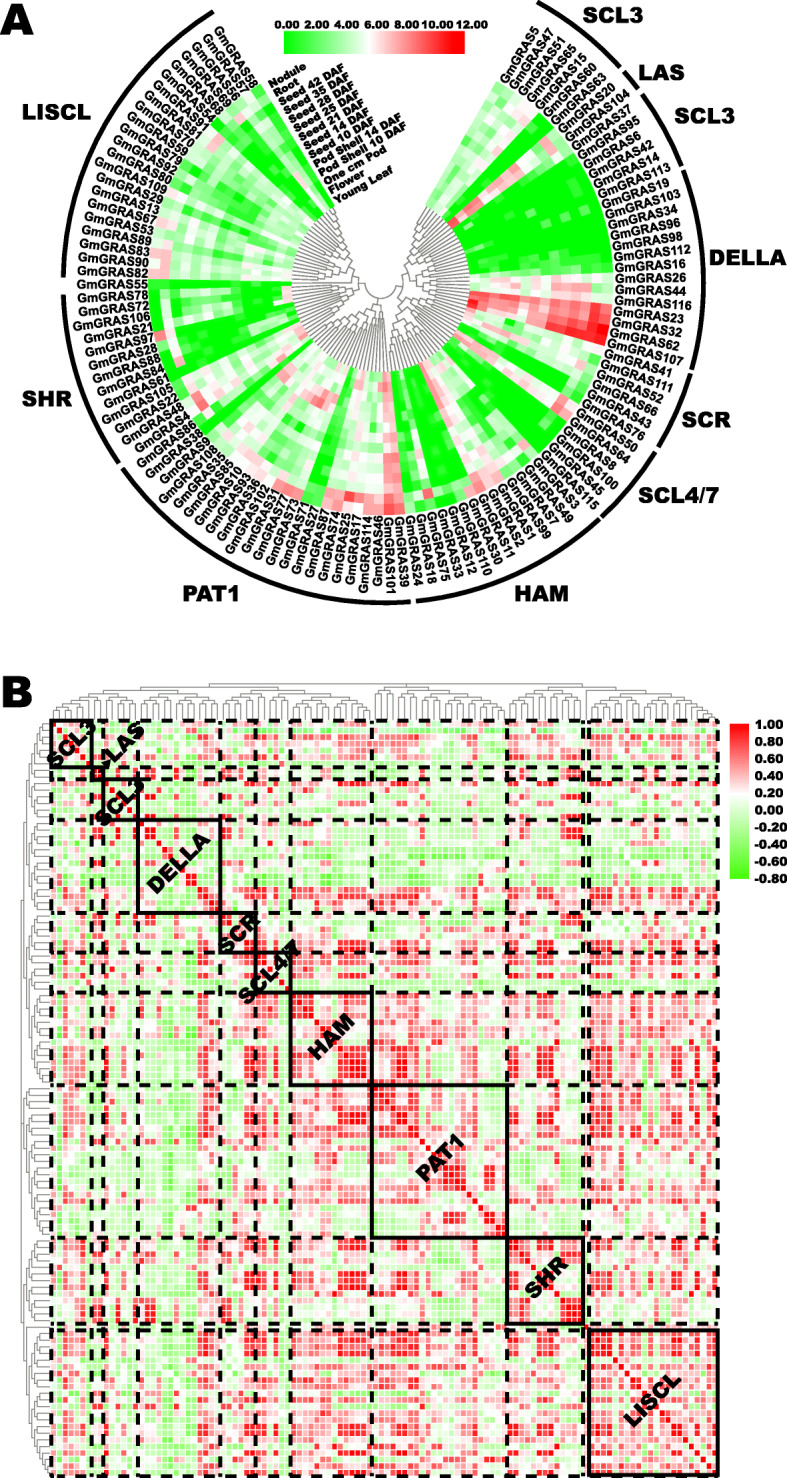
Expression profiles of the *GmGRAS* genes in various tissues during soybean plant developments. **a** Phylogenetically clustered expression profiles of soybean *GRAS* genes in various tissues during soybean developments based on the public transcriptome data. The RPKM values were displayed for gene expression levels and were Log_2_ normalized to depict the heatmap. DAF: days after flowering. **b** Gene expression correlation heatmap of the expressed *GmGRAS* genes in various tissues during soybean development. Red: positively correlated; green: negatively correlated

To investigate the expression profiles of the *GmGRAS* gene members during soybean different developmental stages in diverse tissues, we extracted and analyzed the transcript levels of the *GmGRAS* genes in young leaf, flower, one cm (centimeter) pod, pod shell 10 DAF (days after flowering), pod shell 14 DAF, seed 10 DAF, seed 14 DAF, seed 21 DAF, seed 25 DAF, seed 28 DAF, seed 35 DAF, seed 42 DAF, root and nodule [41]. As a result, 114 *GmGRAS* genes (except for *GmGRAS40*, *GmGRAS94* and *GmGRAS117*) were recruited (Additional file 11: Table S10). Moreover, the expression data of 114 *GmGRAS* genes were Log_2_ normalized to depict a heatmap of *GmGRAS* genes expression profiles in various tissues (Fig. 9a). According to Fig. 9a, the different *GmGRAS* gene subfamilies displayed distinct expression patterns. For instance, most *GmGRAS* genes in the LISCL, SHR and SCL3 subfamilies exhibited low gene expressions in all tissues, however, some genes (*GmGRAS67*, *GmGRAS82* and *GmGRAS83* in the LISCL subfamily; *GmGRAS97* in the SHR subfamily; *GmGRAS15* and *GmGRAS65* in the SCL3 subfamily) showed relatively high expression levels in root and nodule. Notably, nine genes (*GmGRAS39* and *GmGRAS101* in the PAT1 subfamily; *GmGRAS11* in the HAM subfamily; *GmGRAS50* and *GmGRAS64* in the SCL4/7 subfamily; *GmGRAS23*, *GmGRAS32*, *GmGRAS62* and *GmGRAS107* in the DELLA subfamily) presented high expressions in all tissues, which may indicate their crucial functions during soybean plant developments. Interestingly, there were two gene members (*GmGRAS20* and *GmGRAS104*) in the LAS subfamily, and both of them showed high expressions in tissues of leaf, flower, pod, root and early development stages of seed. Besides, most *GmGRAS* genes exhibited relatively superior gene expressions in the root compared with other tissues.

Additionally, we analyzed and calculated the expression correlation coefficients between the 114 *GmGRAS* genes in Table S11 (Additional file 12). A heatmap was depicted based on the correlation coefficients and the heatmap was further clustered by different GmGRAS subfamilies (Fig. 9b). As is shown in Fig. 9b, the correlation heatmap was divided into diverse blocks with the dotted lines according to the GmGRAS subfamilies. Thus, the correlations among different subfamilies were displayed. To emphasize the inter-correlations of each GmGRAS subfamily member, we enclosed the subfamilies with the solid boxes and labeled their names in bold. Most *GmGRAS* genes showed positive correlations with the members of internal or external subfamilies. Whereas, considerable GmGRAS members in the DELLA subfamily tended to exhibit comparatively independent or presented negative correlations with other GmGRAS members. Overall, these results indicated that the functions of *GmGRAS* genes may be widely correlated and varied in soybean tissues.

### Expression profiles of *GmGRAS* genes during saline and dehydration stresses and gene expression correlation analyses

**Fig. 10:**
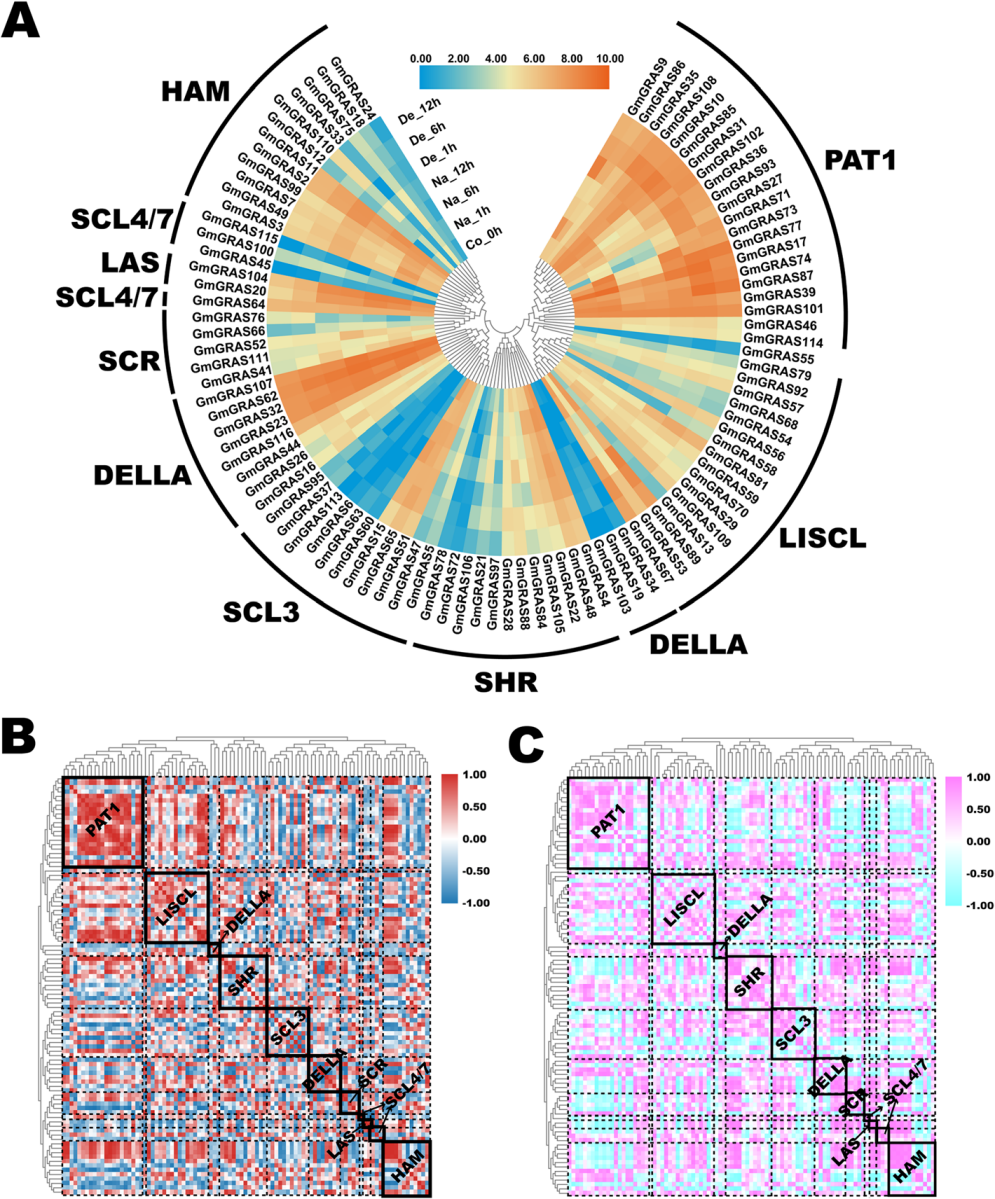
Expression profiles of the *GmGRAS* genes in the soybean root during saline and dehydration stresses. **a** Phylogenetically clustered expression profiles of soybean *GRAS* genes in the soybean root during saline and dehydration stresses. The RPKM values were displayed for gene expression levels and were Log_2_ normalized to depict the heatmap. Na: saline stress; De: dehydration stress; Co: control. **b** Gene expression correlation heatmap of the expressed *GmGRAS* genes in the soybean root during saline stress. Red: positively correlated; dark blue: negatively correlated. **c** Gene expression correlation heatmap of the expressed *GmGRAS* genes in the soybean root during dehydration. Purple: positively correlated; light blue: negatively correlated

Previous studies reported that the *GRAS* genes were broadly responsive to various abiotic stresses [4, 19, 42]. In the present study, we explored the *GmGRAS* gene expression profiles in soybean root during saline and dehydration stresses [43]. As a result, 95 members of the identified *GmGRAS* genes were extracted, and relevant expression information was listed in Table S12 (Additional file 13). Based on the Log_2_ normalized expression data, a heatmap was depicted (Fig. 10a). As is shown in the figure, there were four-time points (0 h, 1 h, 6 h and 12 h)  during the abiotic stresses. And the subfamilies both contained the high expression and low expression *GmGRAS* genes. In particular, most *GmGRAS* genes in the PAT1 subfamily were at relatively high expression levels. Generally, genes that categorized in the same hereditary clades exhibited similarly high or low transcript levels during saline and dehydration stresses.

Moreover, we carried out the gene expression correlation analyses  during saline and dehydration stresses in Table S13 (Additional file 14) and Table S14 (Additional file 15). And two correlation heatmaps were built based on the acquired correlation coefficient matrixes (Fig. 10b and Fig. 10c). Overall, the positive and negative correlations were universally found and interlaced among the GmGRAS members of internal or external subfamilies both  during saline and dehydration stresses. However, the gene expression correlation patterns were distinct during the saline and dehydration stresses. For instance, GmGRAS members in the PAT1 and the SCL3 subfamilies displayed different correlation patterns during saline and dehydration stresses. In all, the different *GmGRAS* gene members were extensively correlated and diversely expressed during saline and dehydration stresses.

### Expression profiles of *GmGRAS* genes in soybean embryonic axes during seed germination and gene expression correlation analyses

**Fig. 11:**
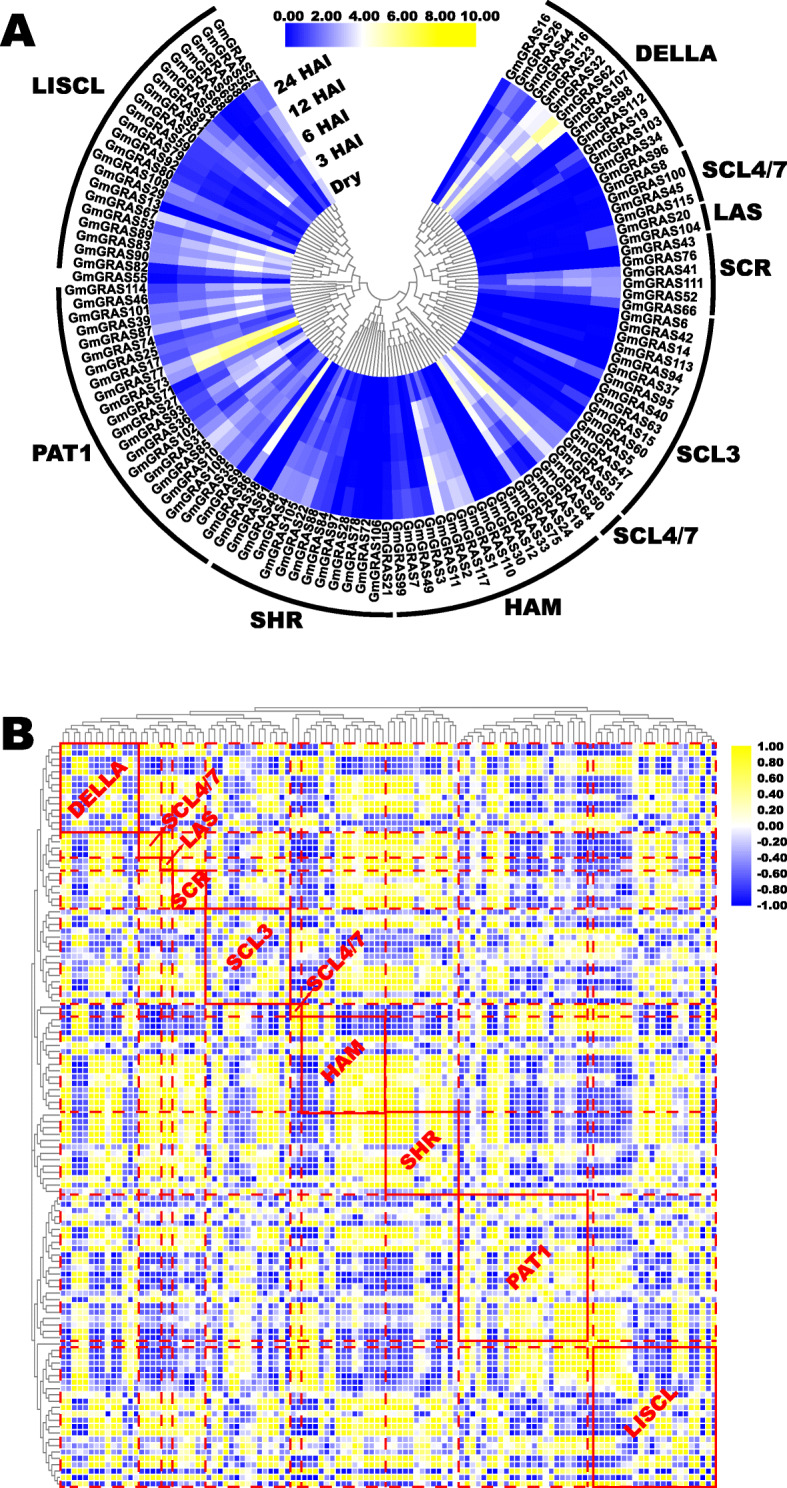
Expression profiles of the *GmGRAS* genes in the soybean embryonic axes during seed germination. **a** Phylogenetically clustered expression profiles of soybean *GRAS* genes in the soybean embryonic axes during germination based on the reported transcriptome data. The FPKM values were displayed for gene expression levels and were Log_2_ normalized to depict the heatmap. HAI: hours after imbibition. **b** Gene expression correlation heatmap of the expressed *GmGRAS* genes in the soybean embryonic axes during seed germination. Yellow: positively correlated; blue: negatively correlated

Seed germination is crucial that influence crop yield and quality. High-vigor soybean embryonic axes were essential to ensure the germination rate [44]. Previous studies paid close attention to the soybean embryonic axis and demonstrated its roles in soybean seed germination from different perspectives [45, 46]. Besides, the *GRAS* family genes were also profoundly affected the seed germination [15]. Hence, we analyzed the relevant expression profiles of the *GmGRAS* genes during soybean germination (Additional file 16: Table S15) [47]. The extracted expression data were Log_2_ normalized to construct a gene expression heatmap (Fig. 11a). As is shown in Fig. 11a, five-time points (dry, 3-HAI (hours after imbibition), 6-HAI, 12-HAI and 24-HAI) were investigated in soybean germination processes. Notably, some soybean *GRAS* genes had comparatively high gene expressions and displayed time-preference. For instance, *GmGRAS73* and *GmGRAS77* in the PAT1 subfamily showed high transcriptional levels at the first four-time points and low expressions at 24-HAI. In contrast, the expression of *GmGRAS62* in the DELLA subfamily continuously up-regulated and displayed high expression at 24-HAI. Moreover, the extracted expression data were further utilized to construct a heatmap, which was row-scaled with the zero-to-one method to show the expression pattern of each *GmGRAS* gene during the seed germination (Additional file 17: Fig. S2). In general, different *GmGRAS* gene members exhibited various expression patterns.

Concomitantly, we conducted the gene expression correlation analyses in Table S16 (Additional file 18) and built the heatmap according to the correlation coefficients of gene expressions (Fig. 11b). As is shown in Fig. 11b, the heatmap was phylogenetically clustered and separated into diverse subunits by red dotted lines to show the correlations among the members of different GmGRAS subfamilies. Moreover, the members of the specific subfamily were highlighted in red solid boxes and annotated with respective subfamily names. On the whole, the *GmGRAS* genes showed positive or negative correlations both internal and external throughout different GmGRAS subfamilies. In conclusion, the identified *GmGRAS* genes were broadly correlated and differently expressed during soybean seed germination.

### Soybean seed germination assay and quantitative RT-PCR analyses of the representative *GmGRAS* genes

**Fig. 12:**
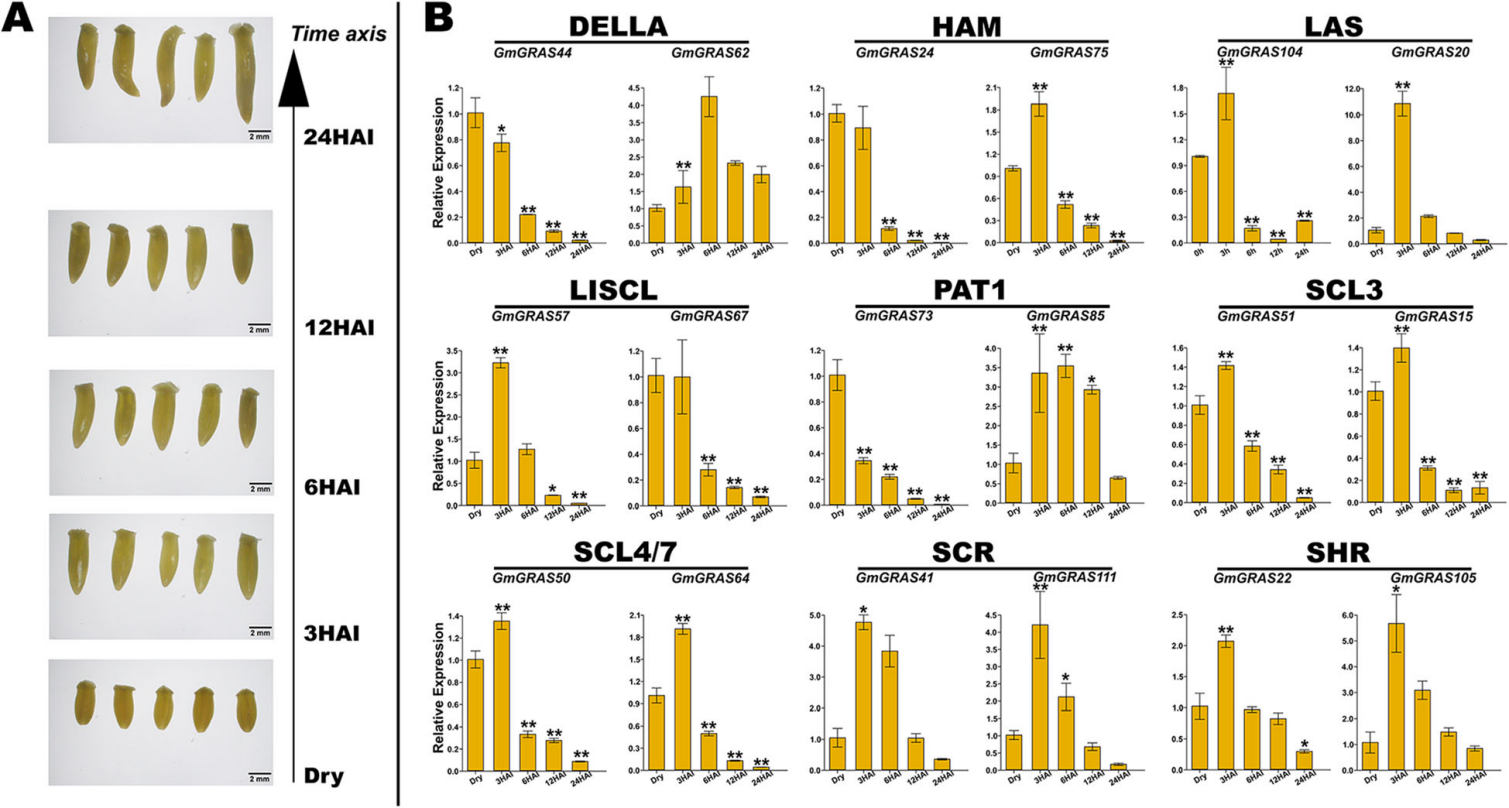
Soybean seed germination experiment and the quantitative PCR analyses of 18 selected *GmGRAS* genes in soybean embryonic axes during seed germination. **a** Photographs of soybean embryonic axes at 0- (Dry), 3-, 6-, 12- and 24-HAI. The size of the soybean embryonic axes can be estimated by using the 2-mm scale at the right bottom. **b** Expression patterns of the selected *GmGRAS* genes in soybean embryonic axes during germination. Data were normalized to the *GmActin* gene, and vertical bars indicated the standard deviations. The values referred to the mean ± standard deviation (SD) of three independent biological replicates. Asterisks manifested the corresponding genes significantly up- or down-regulated compared with those in the dry soybean embryonic axes (* *P* < 0.05, ** *P* < 0.01, Student’s *t*-test)

For the soybean seed germination assay, five representative soybean embryonic axes from each time point were arrayed by the time axis in Fig. 12a. The sampled soybean embryonic axes were carried out the RNA extraction and the quantitative RT-PCR experiment. To further investigate the potential regulating roles of *GmGRAS* genes during soybean seed germination, 18 representative *GmGRAS* genes, whose expression levels were relatively high across different time points (Additional file 16: Table S15), were carefully selected from the identified soybean *GRAS* genes. The selected genes contained two gene members with diverse expression patterns (Additional file 17: Fig. S2) from each *GmGRAS* gene subfamily to cover the differently expressed *GmGRAS* genes as representative as possible. The specific primers of representative *GmGRAS* genes for the quantitative RT-PCR assay were list in Table S17 (Additional file 19). According to the results of the quantitative RT-PCR experiment, the selected genes in distinct *GmGRAS* gene subfamilies tended to exhibit different expression patterns (Fig. 12b). Whereas, the selected genes in some subfamilies displayed similar expression patterns. For instance, the expression levels of *GmGRAS41* and *GmGRAS111* in the SCR subfamily as well as *GmGRAS22* and *GmGRAS105* in the SHR subfamily were up-regulated from 0-HAI (dry) to 3-HAI and were gradually down-regulated from 6-HAI to 24-HAI. Besides, different gene expression patterns also existed in the same subfamily. For example, the expression level of *GmGRAS44* in the DELLA subfamily was consecutively down-regulated from 0-HAI to 24-HAI. In contrast, the expression level of *GmGRAS62* was up-regulated from 0-HAI to 6-HAI and was drown-regulated from 12-HAI to 24-HAI. Conclusively, the expression patterns of the representative genes may outline the diverse *GmGRAS* gene expression tendencies, which highlight the regulations of *GmGRAS* genes in soybean embryonic axes during seed germination.

## Discussion

The GRAS proteins are plant-specific and play important roles in diverse plant developmental and physiological processes. Recently, the genome-wide identifications of GRAS members in different species have been gradually realized with the rapid development of whole-genome sequencing technologies. Nevertheless, associated studies on the soybean *GRAS* gene family are still lacking. In the current research, we identified and carried out a comprehensive investigation on the GRAS transcription factors in soybean, including their phylogenetic relations, gene structures, conserved domains, motif patterns, gene chromosomal distributions, gene duplications, synteny analyses and gene evolutionary analyses. Besides, the expression patterns of the *GmGRAS* genes in various tissues, under saline and dehydration stresses and during seed germination processes were also explored.

In this investigation, 117 *GmGRAS* genes were identified. The GRAS members in soybean exceed those in Arabidopsis (32 members) [8], castor beans (48 members) [25], tomato (53 members) [48] and rice (60 members) [8]. This result was also paralleled to the genome size of the species, which may indicate the positive correlations between specific genome size and the number of GRAS members. Phylogenetic analyses divided the GmGRAS proteins into nine subfamilies based on the classifications on AtGRAS (Fig. 1). The GRAS members categorized in the same subfamily or clade may suggest their similar functions in different species.

Previous research demonstrated that the plant GRAS family originated from the prokaryotic genome and horizontally transferred along with gene duplications [49]. Soybean is an ancient polyploid and has a highly duplicated genome, with approximately 75% of the genes present occurring in multiple copies [50]. According to Table S6 (Additional file 7), apart from tandem duplications, considerable GmGRAS family members were derived from segmental duplication events. Our results verified that both the segmental and tandem duplications were important contributors to the expansion of the *GRAS* gene family. Besides, introns were speculated crucial in plant evolutions, which preferred to raise at the earlier stages of gene expansion and gradually lost over time [51, 52]. Nonetheless, there were also exceptions that introns could be larger during evolutions and were considered to be a necessary way to acquire new gene functions [19, 53]. As is shown in Fig. 2, most detected *GmGRAS* gene members lacked introns, which parallel to the early reports on the conserved characteristics of *GRAS* genes [4, 7]. Notably, most PAT1 subfamily members (16 members) have introns in 5′ UTRs (untranslated regions), which may indicate the PAT1 subfamily is a newly evolved GRAS clade in the soybean genome. The specific domains or motifs of the GRAS proteins ensured the features in protein interaction as well as DNA binding modifications [54]. In the current study, we comprehensively classified the MEME-motifs into the five conserved motifs at the C-terminal of GRAS proteins (Fig. 3b and Fig. 3c). And the MEME-motifs in the five conserved C-terminal motifs fluctuated with diverse subfamilies, which may reflect the distinct biological functions of different GmGRAS subfamily members. In all, the consistencies and divergences of the structures among the GmGRAS members may directly or indirectly illustrate their functional similarities and disparities.

In this investigation, the identified *GmGRAS* genes unevenly distributed on 20 chromosomes and one scaffold of the soybean genome (Fig. 4). Notably, Chr11 contained the most *GmGRAS* genes (16 genes), which were mainly originated from the DELLA, HAM, LISCL, SCR, SHR, SCL3 and SCL4/7 subfamilies (Additional file 1: Table S1). In an early study, Sun et al. elaborately summarized the functional polymorphism of plant GRAS domains: the DELLA domain was associated with transcription co-activation, light signalling, gibberellic acid or jasmonic acid signalling, integrator of abiotic stresses, auxin and ethylene signals; the HAM domain was related to nodulation signalling, transcription co-activation in response to auxin, shoot meristem maintenance; the LISCL domain was relevant to transcription activation or co-activation in response to different signals; the SCR domain participated in root or shoot radial patterning; the SHR domain was linked to transcriptional regulation, root or shoot radial patterning and nodulation signalling; the SCL3 domain can act with DELLA and SHR/SCR to mediate cell elongation in root endodermis; the SCL4/7 domain was the transcription regulator in response to environmental stresses [55]. Besides, among the 16 genes, *GmGRAS48* from the SHR subfamily, *GmGRAS50* from the SCL4/7 subfamily, *GmGRAS51* from the SCL3 subfamily, *GmGRAS52* from the SCR subfamily, *GmGRAS53* and *GmGRAS56* from the LISCL subfamily and *GmGRAS62* from the DELLA subfamily showed relatively high gene expressions in multiple tissues or organs throughout various developmental stages (Additional file 11: Table S10). Taken together, we speculated that some key *GmGRAS* genes on Chr11 may take potential roles in regulating soybean plant growth and development.

To explore the evolutionary relationships of GRAS members among different species, we recruited four dicotyledons and two monocotyledons and carried out the synteny analyses. As a result, soybean and *Glycine soja* exhibited the best orthologous correlations. Comparably, the GRAS members in soybean and *Oryza sativa* performed the weakest orthologous correlations in the current study. In general, the GRAS members in dicotyledons displayed better synteny with the identified GmGRAS members than those in monocotyledons (Fig. 6). Hence, we speculated that the syntenic correlations among GRAS members may be linked to the evolutionary divergence of the species. Notably, 19 GmGRAS members were found to be syntenic with the GRAS members throughout multiple species (Fig. 7a and Additional file 9: Table S8), which indicated that these orthologous pairs are conserved and may exist before the ancestral divergence [35]. Conclusively, the intersections of the syntenic GRAS members among distinct species may be valuable for conducting relevant explorations on GRAS evolution.

The GRAS members were reported widely participate in regulating plant developments and stress responses [4, 19]. In the present research, we analyzed the *cis*-elements in the promoter regions of the detected *GmGRAS* genes. As is shown in Fig. 8, *cis*-elements that associate with the plant growth and development broadly existed, such as the light responsive element, auxin responsive element, meristem expression element. We also detected abiotic stress correlated *cis*-elements, like defense and stress responsive element, drought inducibility element, low temperature responsive element and wound responsive element. Therefore, *cis*-elements analysis supplied clues for gene function study, especially for relevant genes that respond to different stresses and regulate plant developments.

To seek the expression patterns of *GmGRAS* genes in various tissues, we extracted and analyzed the transcript levels of the identified *GmGRAS* genes in different tissues [41]. As a whole, most *GmGRAS* genes were at relatively low transcription levels, and considerable genes clustered in the same gene subfamilies showed similar expression patterns (Fig. 9a). It is worth noting that some *GmGRAS* genes, like *GmGRAS39* and *GmGRAS101* in the PAT1 subfamily displayed high gene expression levels throughout tissues and developmental stages. These *GmGRAS* genes may be crucial and broadly participate in soybean plant developmental processes. Besides, some *GmGRAS* genes, like *GmGRAS20* and *GmGRAS104* in the LAS subfamily exhibited tissues or developmental stages preferences, which highlighted the spatio-temporal regulations of *GmGRAS* genes during soybean plant growth and developments. Furthermore, according to the gene expression correlation analyses in Fig. 9b, most *GmGRAS* genes were positively correlated with each other, which demonstrated that the *GmGRAS* genes widely cooperated in associated regulations. Previously, the DELLA subfamily members in different plants had been extensively studied, which were the key negative regulators of the GA (gibberellic acid) signal transduction pathways and inhibited plant growth and developments [56, 57]. In this study, most *GmGRAS* genes in the DELLA subfamily showed negative correlations with other *GmGRAS* genes or displayed relative independence. Associated with gene expression profiles of the DELLA subfamily genes in Fig. 9a, we speculated that several key genes in the DELLA subfamily may persistently express and regulate soybean growth and developments. Overall, the identified *GmGRAS* genes exhibited potential functions in regulating soybean plant developments.

GRAS members are relevant to regulating their biochemical activities in response to abiotic stresses [12]. In this study, the roles of some GmGRAS members during saline and dehydration stresses were investigated [43]. Importantly, considerable *GmGRAS* genes throughout different *GmGRAS* gene subfamilies were influenced both by the saline and dehydration stresses. As a whole, the stress responses (high or low gene expression levels) of the identified *GmGRAS* genes presented similarities during saline and dehydration stresses (Fig. 10a), which may highlight the roles of key *GmGRAS* genes in response to the abiotic stresses. For instance, most *GmGRAS* genes in the PAT, LAS and DELLA subfamilies showed high expressions during saline and dehydration stresses (Fig. 10a). Notably, DELLA proteins were reported to play an important role in regulating plant stress tolerance [57], and our investigation further verified the previous findings. Whereas, the gene expression correlation patterns during saline and dehydration stresses were distinctly different (Fig. 10b and Fig. 10c). Hence, we speculated that functional *GmGRAS* genes may broadly cooperate in response to saline and dehydration stresses under different mechanisms.

Particularly, we investigated the expression profiles of *GmGRAS* genes in soybean embryonic axes during seed germination [47]. Interestingly, most *GmGRAS* genes relatively low expressed. However, there were also some high expressed *GmGRAS* genes in different GmGRAS subfamilies (Fig. 11a). And the *GmGRAS* genes displayed diverse expression patterns during the seed germination (Additional file 17: Fig. S2). Importantly, as is shown in Fig. 11b, distinct *GmGRAS* genes were universally correlated, which indicated the functional *GmGRAS* genes may broadly interact and concertedly affect soybean seed germination. Furthermore, we selected 18 representative *GmGRAS* genes and carried out the quantitative RT-PCR analyses. Compared to the reported expression patterns of *GmGRAS* genes in Fig. S2 (Additional file 17), *GmGRAS24*, *GmGRAS41*, *GmGRAS44*, *GmGRAS50*, *GmGRAS51*, *GmGRAS57*, *GmGRAS64*, *GmGRAS67*, *GmGRAS73* and *GmGRAS75* that validated by the quantitative RT-PCR assay showed fundamental uniformity (Fig. 12b). Whereas, *GmGRAS15*, *GmGRAS20*, *GmGRAS22*, *GmGRAS62*, *GmGRAS85*, *GmGRAS104*, *GmGRAS105* and *GmGRAS104* displayed different expression patterns from those of the reported transcriptome data [47]. For instance, in the transcriptome data, the transcript levels of *GmGRAS62* was gradually up-regulated from 0-HAI to 24-HAI (Additional file 17: Fig. S2). However, in the quantitative RT-PCR assay result, the expression levels *GmGRAS62* was first up-regulated from 0-HAI to 6-HAI then was down-regulated from 6-HAI to 24-HAI (Fig. 12b). For the soybean seed gemination assay in the current research, we added the seed presoaking process compared to the early study [47], which accelerated soybean seed imbibition. And this may cause the preceding expression of relevant *GmGRAS* genes. Another possible cause may due to the differences between testing soybean varieties in the present study (cv. ‘Williams 82’) and the previous research (cv. ‘BRS 284’) [47]. In summary, *GmGRAS* genes have potential regulatory roles during soybean seed germination.

This study provided a systematic investigation of *GRAS* genes in soybean, which may be beneficial to gain insights into their biological functions. However, the current study only provided a preliminary characterization of *GmGRAS* genes and further functional validation should be carried out to understanding the different roles of *GmGRAS* genes in various biological processes.

## Conclusions

In this study, we identified 117 *GmGRAS* genes in soybean. These *GmGRAS* genes unevenly located on 20 chromosomes and one scaffold in the soybean genome. The identified GmGRAS members were further classified into nine GmGRAS subfamilies. Gene structure analyses turned out that most *GmGRAS* genes lack introns, suggesting that the structures of *GmGRAS* genes were highly conserved. Conserved domain and motif pattern analyses showed that the GRAS members in the same subfamily or clade displayed broadly similarities, which may indicate their parallel gene functions. Moreover, the emergence of new *GmGRAS* genes mainly drove by gene duplications, and segmental duplication events took the lead in *GmGRAS* gene family expansion. Besides, *cis*-element and gene expression analyses revealed the potential regulations of the identified *GmGRAS* genes in various tissues, during saline and dehydration stresses and during soybean seed germination processes. In all, we comprehensively investigated the characteristics of *GRAS* genes in soybean, and the results provided valuable clues for understanding the gene biological functions and future studies on *GmGRAS* genes.

## Methods

### Mining of GRAS family members in soybean

For the identification of GRAS genes in soybean (soybean *Wm82.a2.v1* genome version), we obtained all the AtGRAS protein sequences as the query sequences from the TAIR database (https://www.arabidopsis.org/) [4, 58]. The soybean genome and genome annotation files were downloaded from Phytozome v12. 1.6 database (https://phytozome.jgi.doe.gov/pz/portal.html). Based on these data, we extracted the most representative GmGRAS member sequences by using TBtools software [59], and GmGRAS protein sequences were further queried and verified in the NCBI protein database by BLASTp (https://blast.ncbi.nlm.nih.gov/Blast.cgi? PROGRAM = blastp&PAGE_TYPE = BlastSearch&LINK_LOC = blasthome). For the domain composition analyses, we used the NCBI-Conserved Domain database (https://www.ncbi.nlm.nih.gov/Structure/cdd/wrpsb.cgi). Proteins that lack GRAS associated domains were manually deleted. Moreover, protein sequences with obvious errors in their gene length or less than 100 aa (amino acid) were also removed. The ExPASy website (http://expasy.org/tools/) was employed for evaluations of molecular weight (MW), isoelectric point (pI) and amino acid numbers of the identified GmGRAS proteins. For *GmGRAS* gene subcellular localization predictions, we used the CELLO (http://cello.life.nctu.edu.tw/) online tools.

### Phylogenetic analyses and classifications of the GmGRAS proteins

The GRAS protein sequences of Arabidopsis and soybean were together aligned by using the muscle method of MEGA 7.0 (https://www.megasoftware.net/) with the default parameters. The aligned sequences were followed by the neighbor-joining (NJ) method to build the phylogenetic tree, with the following parameters: Poisson model, pairwise deletion, and 1000 bootstrap replications [35]. The identified GmGRAS proteins were further categorized into different subfamilies based on the records of AtGRAS subfamily members in the TAIR database (https://www.arabidopsis.org/). The modified phylogenetic tree was depicted by FigTree v1.4.3 (http://tree.bio.ed.ac.uk/software/figtree/) and Adobe Illustrator CC 2019 (https://www.adobe.com/products/illustrator/free-trial-download.html).

### Gene structures and conserved motif analyses

The gene structures were depicted by TBtools [59] with the GFF3 file of the soybean genome. The conserved motifs scanning of GmGRAS proteins were conducted by MEME v5.1.1 (http://meme-suite.org/tools/meme) with 20 MEME-motifs shown in the result. The visualization and the Seq Logos of the MEME-motifs were created by TBtools. Referring to the classifications of Quan et al. on the GRAS C-terminal conserved motifs, the identified MEME-motifs were further categorized into the specific LHR I, VHIID, LHR II, PFYRE and SAW motifs [34]. The output graphs were modified by Adobe Illustrator CC 2019.

### *GmGRAS* gene chromosomal locations, duplications and synteny analyses

The chromosomal locations and duplications of *GmGRAS* genes were mapped according to the available soybean genome information on Phytozome and displayed by the TBtools software [59]. The gene density information of each chromosome or scaffold was calculated by TBtools. To explore the synteny relationships of the orthologous *GRAS* genes among soybean and other species, we additionally downloaded the genome data and the gene annotation files of *Arabidopsis thaliana* (TAIR annotation release 10), *Glycine soja* (V1.1), *Vigna unguiculata* (V1.1), *Solanum lycopersicum* (ITAG3.2), *Oryza sativa* (MSU annotation release 7.0) and *Sorghum bicolor*) (V3.2) from Phytozome. The syntenic analyzing graphs were constructed by using the Dual Synteny Plotter function in TBtools. The Venn diagram of the syntenic *GRAS* genes throughout diverse species was depicted by TBtools. Non-synonymous substitution (Ka) and synonymous substitution (Ks) of the duplicated *GRAS* genes were calculated by TBtools. The output graphs were modified by Adobe Illustrator CC 2019.

### *Cis*-element analyses of *GmGRAS* genes

The upstream 2000 bp sequences of the identified *GmGRAS* genes were extracted by TBtools [59]. Then the extracted sequences were submitted to PlantCARE website (http://bioinformatics.psb.ugent.be/webtools/plantcare/html/) to predict the *cis*-elements in promoter regions. The diagram of *cis*-elements of *GmGRAS* genes was displayed by TBtools and was modified by Adobe Illustrator CC 2019.

### Expression profile analyses of *GmGRAS* genes

The transcriptional levels of the identified *GmGRAS* genes in different tissues were retrieved from the transcriptome data on SoyBase (https://soybase.org/soyseq/) [41], which were assessed by the RPKM (reads per kilobase million) values. The expression profiles of *GmGRAS* genes during saline and dehydration stresses were obtained by the RPKM values from the published research data [43]. The transcriptional levels of the *GmGRAS* genes in soybean embryonic axes during germination were extracted from an early study of Bellieny-Rabelo et al., which were scored by the FPKM (fragments per kilobase million) values [47]. Heatmaps of the *GmGRAS* gene expressions were illustrated based on the Log_2_ normalized RPKM or FPKM values by using TBtools [59]. To explore the *GmGRAS* gene expression correlations, both the extracted RPKM and FPKM values were submitted and calculated on Omicshare online tools (https://www.omicshare.com/tools/Home/Soft/getsoft). Besides, the correlation heatmaps of *GmGRAS* gene expressions were phylogenetically clustered and depicted by TBtools. The output graphs were modified by Adobe Illustrator CC 2019.

### Plant material and soybean seed germination assay

Soybean cultivar Williams 82, a well-known soybean genome referencing variety, was used in this study. Three independent soybean plants were randomly selected and harvested the seeds for three independent biological replicates in this study. All the testing soybean seeds were harvested in the autumn of 2019 at the Dangtu Experimental Station, National Center for Soybean Improvement, Nanjing Agricultural University, Dangtu, Anhui, China. Referring to the previous study, we focused five-time points during soybean seed germination: dry, 3-HAI (hours after imbibition), 6-HAI, 12-HAI and 24-HAI [47]. Importantly, we adopted the germination pouches (medium size, 18 cm height and 12.5 cm width, PhenoTrait Technology Co., Ltd.) to conduct the soybean seed germination experiment. For each time point, 40 healthy seeds were selected from the independent soybean plant harvesting seeds. And the seeds were placed in a 40 °C drying oven for 3 days to ensure the uniformity of seed moisture. The selected seeds were then disinfected by 0.05% potassium permanganate solution for 5 min and washed with deionized water. According to the instruction book of the germination pouch, the disinfected soybean seeds were soaked in deionized water for 2 hours to accelerate seed imbibition and ensure seed germination. Next, we placed eight seeds in each germination pouch and added 15 mL deionized water. Then germination pouches were put on the germination pouch shelf and placed in the dark in a temperature-controlled incubator at 25 °C. Embryonic axes were carefully separated from cotyledons for RNA extraction at each time point. Five representative seed embryonic axes at each time point were photographed by using a SPOT-RT digital camera (Diagnostic Instruments, Sterling Heights, MI) and an OLYMPUS SZ61 stereomicroscope (Olympus, Melville, NY, USA).

### RNA isolations and quantitative RT-PCR analyses

To validate the expression patterns in embryonic axes during soybean seed germination, we selected 18 representative *GmGRAS* genes for the quantitative RT-PCR analyses. Three independent biological replicates contained three independent plants were used for quantitative RT-PCR. The specific quantitative RT-PCR primers of the selected *GmGRAS* genes were designed by Primer Premier 5 and listed in Table S17 (Additional file 19). Referring to the former research of Bellieny-Rabelo et al., 20 healthy seed embryonic axes were randomly selected from the 40 embryonic axes of each independent plant each time point for total RNA isolations [47]. The total RNA was extracted by using the RNAprep pure plant kit (TIANGEN, Beijing, China) from the frozen embryonic axes. All RNA was analyzed by electrophoresis and then quantified with a Nanodrop ND-1000 spectrophotometer (Nanodrop, Wilmington, DE, USA). The HiScript II 1st Strand cDNA Synthesis Kit (Vazyme Biotech, Nanjing, China) was adopted to remove the genomic DNA and to convert the total RNA to cDNA [60]. The SYBR qPCR Master Mix (Vazyme Biotech, Nanjing, China) was adopted to conduct the quantitative RT-PCR assay on a BioRad CFX96 real-time system [60]. The housekeeping *GmActin* gene was determined as an internal control. Triplicate quantitative assays were performed on each cDNA sample and analyzed by a 2^−△△CT^ method [61].

### Statistical analyses

Student’s *t*-test was performed by using by Graphpad Prism 8 (https://www.graphpad.com/scientific-software/prism/). Test differences were determined to be significant with a *P*-value cut-off of 0.05. All the error bars were standard deviation (SD) from the independent biological replicates.

## Supplementary information

**Additional file 1: Table S1**. The 117 identified *GmGRAS* genes in this study.

**Additional file 2: Table S2**. Coding sequences and protein sequences of the identified soybean *GRAS* gene members.

**Additional file 3: Table S3**. Corresponding names of 32 *AtGRAS* genes in Arabidopsis.

**Additional file 4: Table S4**. Analyses the motifs in soybean GRAS proteins from the MEME website.

**Additional file 5: Figure S1**. Seq Logos of 20 MEME-motifs for the identified GmGRAS proteins.

**Additional file 6: Table S5**. Gene density of each chromosome or scaffold of the soybean genome.

**Additional file 7: Table S6**. Tandemly and segmentally duplicated *GmGRAS* gene pairs.

**Additional file 8: Table S7**. One-to-one orthologous relationships between the *GRAS* gene members in soybean and the other six species.

**Additional file 9: Table S8**. Non-redundant *GmGRAS* gene IDs associated with the syntenic relationships between soybean and the other six species.

**Additional file 10: Table S9**. *Cis*-element analyses of the *GmGRAS* gene promoter regions.

**Additional file 11: Table S10**. Expression profiles of *GmGRAS* genes in multiple tissues throughout various developmental stages.

**Additional file 12: Table S11**. Pairwise correlation coefficients between different expressed *GmGRAS* genes in various tissues.

**Additional file 13: Table S12**. Expression profiles of *GmGRAS* genes in soybean root during dehydration and salt stresses.

**Additional file 14: Table S13**. Pairwise correlation coefficients between different expressed *GmGRAS* genes during saline stress.

**Additional file 15: Table S14**. Pairwise correlation coefficients between different expressed *GmGRAS* genes during dehydration stress.

**Additional file 16: Table S15**. Expression profiles of *GmGRAS* genes in soybean embryonic axes during seed germination.

**Additional file 17: Figure S2**. Phylogenetically clustered expression patterns of the expressed *GmGRAS* genes in soybean embryonic axes during germination based on the reported transcriptome data. The FPKM value was row-scaled with the zero-to-one method to show the expression pattern of each *GmGRAS* gene during the seed germination.

**Additional file 18: Table S16**. Pairwise correlation coefficients between different expressed *GmGRAS* genes in soybean embryonic axes during seed germination.

**Additional file 19: Table S17**. Sequences of the primers used in this study.

## Data Availability

All data generated or analyzed during this study are included in this published article and its Additional files.

## Abbreviations

*Gm*: *Glycine max*
TFs: Transcription factors
GAI: Gibberellic acid insensitive
RGA: Repressor of GA1–3 mutant
SCR: Scarecrow
LHRI: Leucine-rich region I
LHRII: Leucine-rich region II
*Va*: *Vitis amurensis*
*At*: *Arabidopsis thaliana*
phyA: Phytochrome A
*Mt*: *Medicago truncatula*
aa: Amino acid
MW: Molecular weight
pI: Isoelectric point
NJ: Neighbor-joining
Ka: Non-synonymous substitution
Ks: Synonymous substitution
RPKM: Reads per kilobase million
FPKM: Fragments per kilobase million
HAI: Hours after imbibition
SD: Standard deviation
ORF: Open reading frame
CDS: Coding sequence
Chr: Chromosome
kb: Kilobase
*Gs*: *Glycine soja*
*Vu*: *Vigna unguiculate*
*Sl*: *Solanum lycopersicum*
*Sb*: *Sorghum bicolor*
*Os*: *Oryza sativa*
cm: Centimeter
DAF: Days after flowering
UTRs: Untranslated regions
