# Supplementary figures and images for: Genome-wide identification and characterization of GRAS genes in soybean (Glycine max)
Source: BMC Plant Biol. 2020 Sep 5;20:415. doi: 10.1186/s12870-020-02636-5 (PMC7487615; doi:10.1186/s12870-020-02636-5)

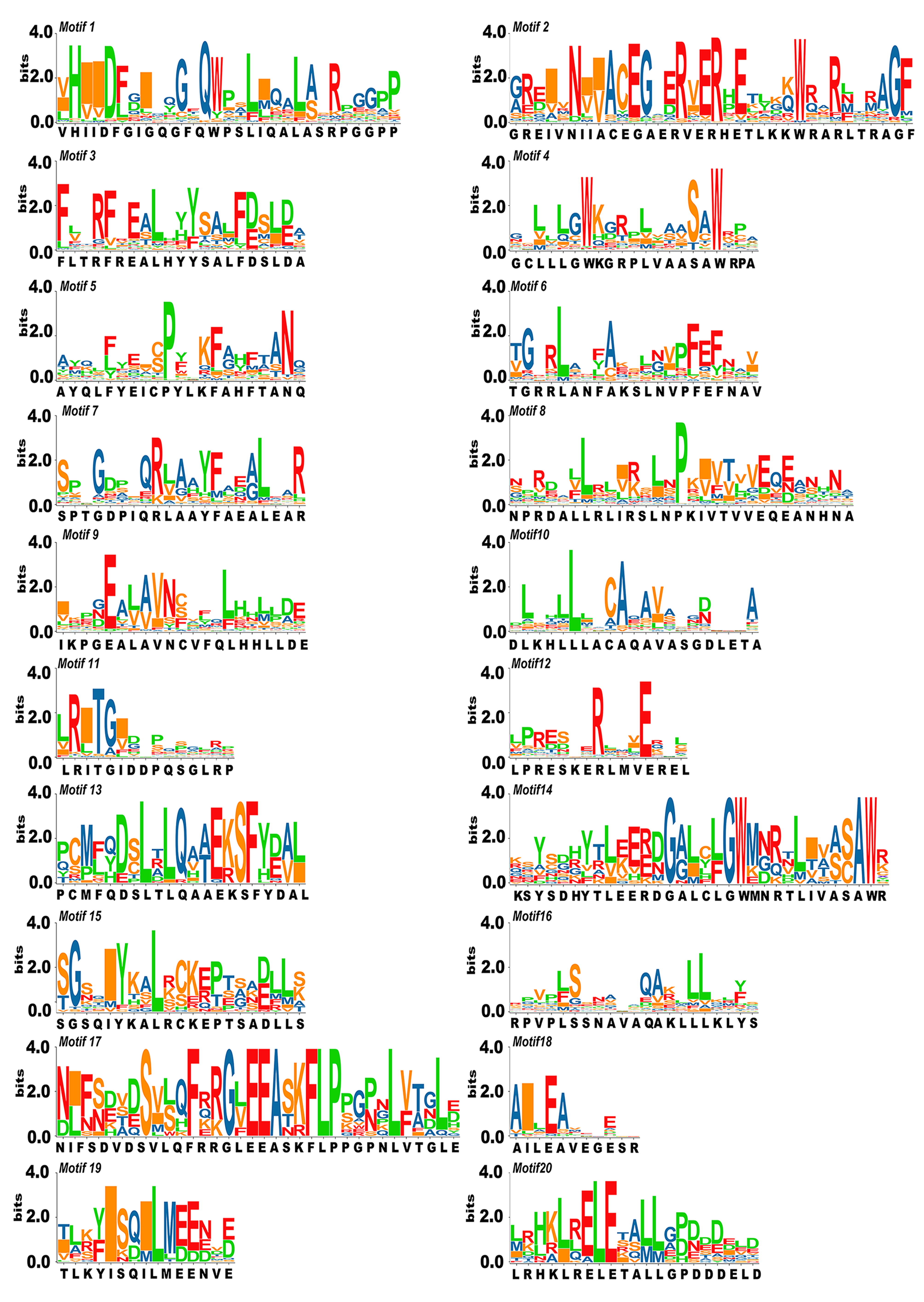

Supplement: Supplementary file 5 — Additional file 5: Figure S1. Seq Logos of 20 MEME-motifs for the identified GmGRAS proteins. [file 12870_2020_2636_MOESM5_ESM.png]

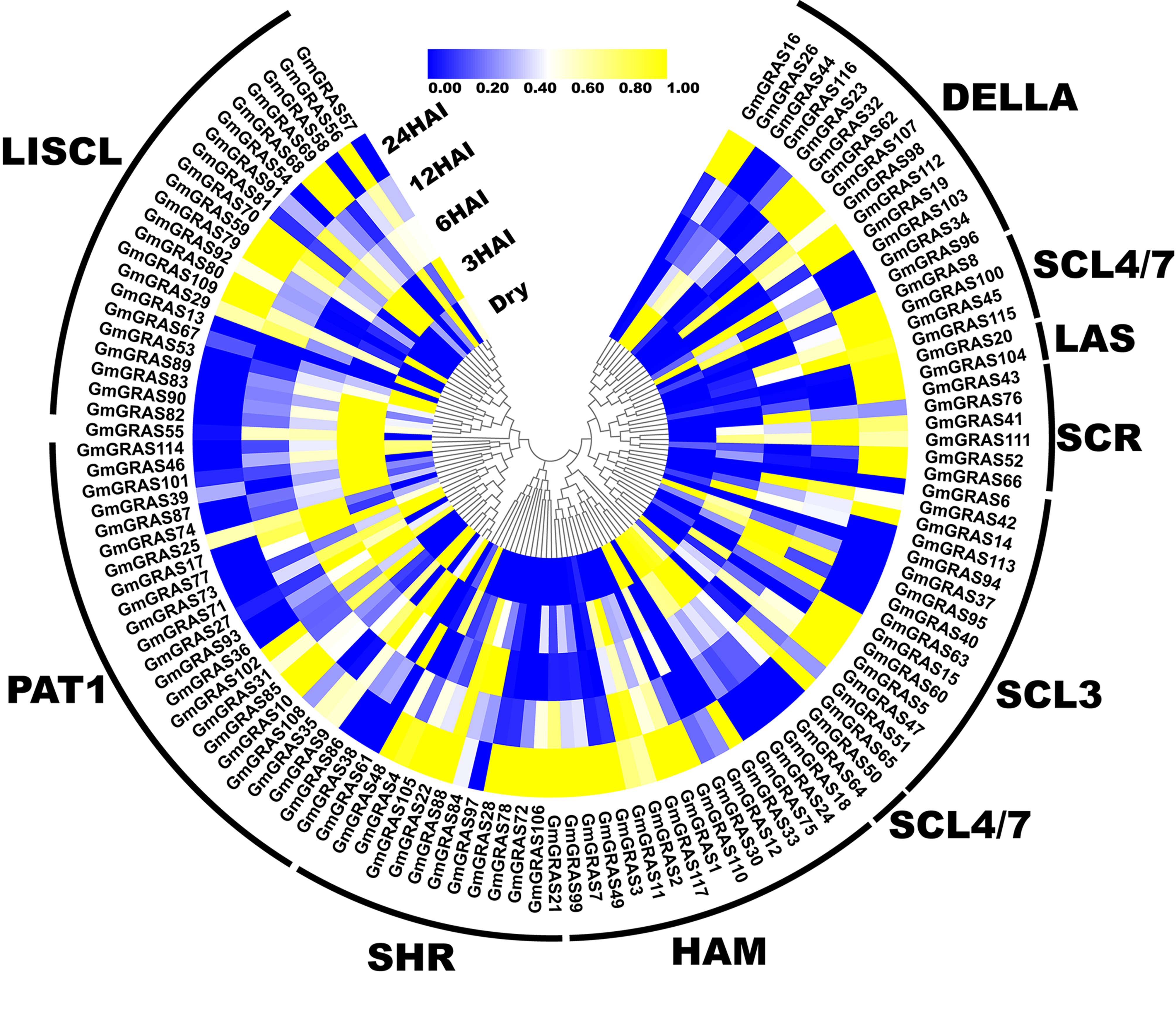

Supplement: Supplementary file 17 — Additional file 17: Figure S2. Phylogenetically clustered expression patterns of the expressed GmGRAS genes in soybean embryonic axes during germination based on the reported transcriptome data. The FPKM value was row-scaled with the zero-to-one method to show the expression pattern of each GmGRAS gene during the seed germination. [file 12870_2020_2636_MOESM17_ESM.png]
